# The impact of a mentalization-based psychotherapy program on borderline personality disorder hospitalizations: a protocol for a 20-year analysis using electronic health records

**DOI:** 10.1192/j.eurpsy.2025.1906

**Published:** 2025-08-26

**Authors:** M. Azevedo, E. Osório, R. Barranha, A. R. Ferreira

**Affiliations:** 1 Faculty of Medicine, University of Porto; 2 Psychiatry Service, Unidade Local de Saúde de São João; 3 Department of Clinical Neurosciences and Mental Health, Faculty of Medicine, University of Porto; 4 CINTESIS@RISE, Faculty of Medicine, University of Porto, Porto, Portugal

## Abstract

**Introduction:**

Borderline personality disorder (BPD) is a severe and complex mental disorder and a major contributor to disability, health-care costs, and societal expenses worldwide. BPD is associated with considerable functional impairment and high healthcare utilization, often co-occurring with other mental disorders such as depression, substance use disorders, and bipolar disorder, further entangling its presentation. Coupled with the high risk of self-harm and suicidal behaviours, this calls for an evidence-based treatment, with a timely intervention to reduce both individual suffering and intensive care utilization. BPD treatment remains challenging. With its neurobiological underpinnings yet to be fully clarified, no medication is specifically approved for BPD, being psychotherapy approaches the treatment of choice. However, since not all patients respond sufficiently to psychotherapy, further research in this area is of utmost importance.

**Objectives:**

To assess the impact of a structured mentalization-based therapy (MBT) program on the number and length of psychiatric hospitalizations of patients with a discharge diagnosis of BPD.

**Methods:**

An observational retrospective study will be conducted using routinely collected health data from adult patients admitted to the inpatient unit in the psychiatry service of the Unidade Local de Saúde de São João (ULSSJ), a University Hospital in Porto, Portugal. Using data from 2003 to 2023, a 20-year analysis will be performed, allowing for the comparison of hospitalizations before and after the program introduction. The study was approved by the Ethics Committee of ULSSJ under the approval n.º 337-23.

**Results:**

The number and length of hospitalizations before and after the implementation of this program will be compared, to evaluate its impact. Findings will be reported using the RECORD guidelines and disseminated via publication in peer-reviewed journals.

**Conclusions:**

With this analysis we expect to enhance the understanding of the impact of psychotherapies on this disorder and, hopefully, demonstrate that implementing a MBT program for BPD patients may lead to a decrease in the number and length of hospitalizations due to BPD, which may be indicative of more effective treatment and lower healthcare costs associated with this disorder.

**Disclosure of Interest:**

None Declared

